# Telmisartan Activates PPARδ to Improve Symptoms of Unpredictable Chronic Mild Stress-Induced Depression in Mice

**DOI:** 10.1038/s41598-017-14265-4

**Published:** 2017-10-25

**Authors:** Yingxiao Li, Kai-Chun Cheng, Keng-Fan Liu, Wen-Huang Peng, Juei-Tang Cheng, Ho-Shan Niu

**Affiliations:** 1 Department of Psychosomatic Internal Medicine, Kagoshima University Graduate School of Medical and Dental Sciences, Kagoshima, 890 Japan; 2 Department of Medical Research, Chi-Mei Medical Center, Yong Kang, Tainan City, 71003 Taiwan; 3 School of Chinese Pharmaceutical Sciences and Chinese Medicine Resources, College of Pharmacy, China Medical University, Taichung City, 40401 Taiwan; 4 Institute of Medical Sciences, Chang Jung Christian University, Gueiren, Tainan City, 71101 Taiwan; 5 Department of Nursing, Tzu Chi University of Science and Technology, Hualien City, 97005 Taiwan

## Abstract

Major depression is a common mental disorder that has been established to be associated with a decrease in serotonin and/or serotonin transporters in the brain. Peroxisome proliferator-activated receptor δ (PPARδ) has been introduced as a potential target for depression treatment. Telmisartan was recently shown to activate PPARδ expression; therefore, the effectiveness of telmisartan in treating depression was investigated. In unpredictable chronic mild stress (UCMS) model, treatment with telmisartan for five weeks notably decrease in the time spent in the central and the reduced frequency of grooming and rearing in open filed test (OFT) and the decreased sucrose consumption in sucrose preference test (SPT) compared with the paradigms. Telmisartan also reversed the decrease in PPARδ and 5-HTT levels in the hippocampus of depression-like mice. Administration of PPARδ antagonist GSK0660 and direct infusion of sh-PPARδ into the brain blocked the effects of telmisartan on the improvement of depression-like behavior in these mice. Moreover, telmisartan enhanced the expression of PPARδ and 5HTT in H19-7 cells. In conclusion, the obtained results suggest that telmisartan improves symptoms of stress-induced depression in animals under chronic stress through activation of PPARδ. Therefore, telmisartan may be developed as a potential anti-depressant in the future.

## Introduction

The chronic and stressful life events are associated with the onset of major depression, which is the most prevalent psychiatric disorder with high morbidity and mortality rates^1^. Efforts to reduce the prevalence of depression continue due to its public health significance. Therefore, the model of unpredictable chronic mild stress (UCMS) was developed to investigate depressive phenomena and drug treatment in animals. Clinical and experimental data have shown that the disturbances in the serotoninergic system and stress play a key role in depressive disorders^2^. Serotonin (5-HT) released from serotonergic terminals is selectively taken up from the synaptic cleft into these terminals via the serotonin transporter (5-HTT)^3^. In depression, the extensive degeneration of serotonergic neurons corresponds to the loss of 5-HTT^4^. Additionally, 5-HTT knockout mice show several behavioral changes, including increased anxiety-like behavior, increased sensitivity to stress, and inhibited exploratory locomotion^5^.

Peroxisome proliferator-activated receptors δ (PPARδ), as one of the receptors in the PPAR nuclear receptor family, is a ligand-activated transcription factor. PPARδ regulates energy metabolism and mitochondrial biogenesis in skeletal muscle^6^. PPARδ shows a widespread brain distribution, it is least two-fold more highly expressed in brain than in muscle^7^. Recently, PPARδ was shown to play an important role in repress stress-induced depressive behaviors^8^ in addition to the regulation of serotonin transporter expression in hipopcampus^9^. Moreover, PPARδ activation also produces neuroprotection and reverses neurodegeneration in Alzheimer’s disease^10,11^, Parkinson’s disease^12^ and Huntington’s disease^13^. Generally, the hippocampus has been widely selected to investigate 5-HTT and PPARδ expression levels, as this brain region has been strongly implicated in the cause and consequences of both depression and chronic stress^14^.

Telmisartan, an angiotensin II type 1 receptor blocker (ARB), is widely used to treat hypertension with the expectation of a decrease in the onset of cardiovascular and cerebrovascular disease. As the most lipophilic agent with the longest half-life among ARBs^15^, telmisartan is known to cross the brain-blood barrier (BBB) for blockade of central AT1 receptors^16^. Telmisartan was identified to play a role in neurological system. Since BBB permeability is increased due to stress^17^, the effect of peripherally administered telmisartan on cerebral function seems sufficient to attenuate the stress-induced cognitive decline. Telmisartan exhibited anti-apoptosis, anti-inflammatory, and antioxidant benefits in the intracerebral hemorrhage rat model^18^. In Parkinson’s disease, telmisartan was reported to protect mouse dopaminergic neurons and inhibit the microglial response^19^. Telmisartan has been recently discovered to activate PPARδ for the promotion of glucose uptake to improve insulin sensitivity and hyperglycemia-induced cardiac fibrosis^20,21^.

In the present study, we investigated the effect of telmisartan on stress-induced depression in animals. In the UCMS mice model, the behavior performances including open filed test (OFT) and the sucrose preference test (SPT) were evaluated. The effect of telmisartan and losartan, a selective AT receptor antagonist, also compared. Moreover, the expression levels of PPARδ and 5-HTT in the hippocampus between vehicle-treated group and telmisartan-treatment group were determined in UCMS mice. To further understanding the relationship between telmisartan and PPARδ, the expression of PPARδ were knockdown or knockout using PPARδ specific antagonist GSK0660 or ShRNA. Additionally, the effects of telmisartan on expressions of PPARδ and 5-HTT were further studied in the H19-7 cell line.

## Results

### Telmisartan ameliorated anxiety- and depression-like behavior in UCMS mice

**Figure 1:**
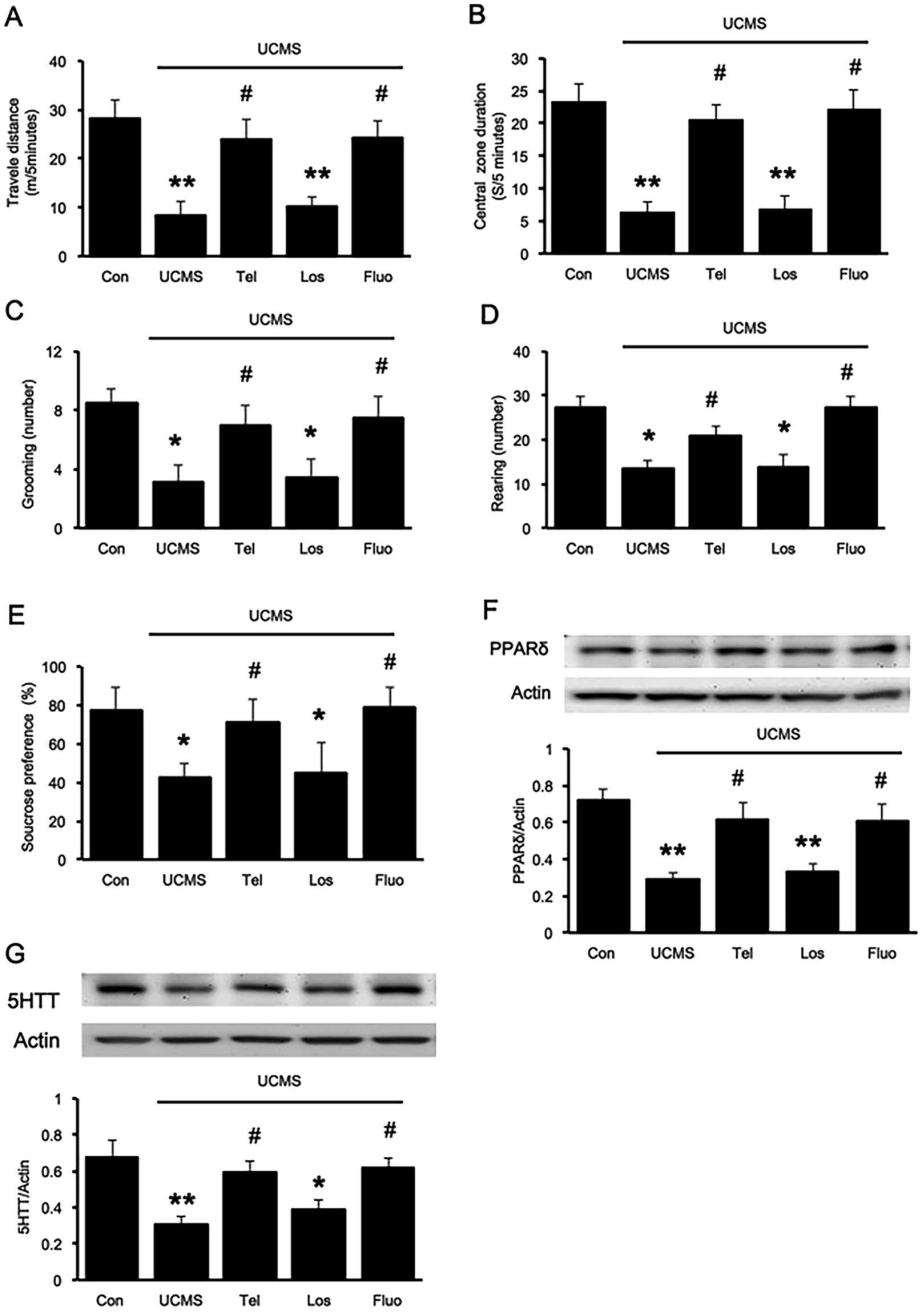
Telmisartan ameliorated the depression-like behavior through the PPARδ pathway in mice receiving UCMS. The animals were allowed to explore the open field arena for 5 min. (**A**) The total distance travelled; (**B**) the time spent in the central; (**C**) frequency of grooming; (**D**) frequency of rearing; (**E**) the percentage of sucrose solution consumed (%) in the sucrose preference test (SPT); (**F**) PPARδ expression levels in the hippocampus; (**G**) 5-HTT expression levels in the hippocampus. Data are presented as the mean ± SEM (n = 8 per group). *p < 0.05 and **p < 0.01 compared to the corresponding control; ^#^p < 0.05 and ^##^p < 0.01 compared to the UCMS group. Full-length blots are presented in Supplementary Fig. S1.

The open field test was performed to evaluate locomotion and anxiety-like behaviors; stress affected the time spent in the center and the periphery. Reduced time spent in the OFT center is used to measure the anxiety-like behavior. Mice that received daily oral administration of telmisartan showed a significant increase in the total distance travelled F_(4,35)_ = 62.311, P < 0.05; Fig. 1A] and time spent in the central and was more likely to explore the environment than the vehicle-treated group [F_(4,35)_ = 84.202, P < 0.05; Fig. 1B]. The number of grooming [F_(4,35)_ = 24.598, P < 0.05] and rearing behavior [F_(4,35)_ = 69.947, P < 0.05] were also markedly suppressed in the UCMS group [Fig. 1C,D]. Additionally, mice with UCMS showed less preference in sucrose intake, but the sucrose consumption was reversed in both telmisartan- and fluoxetine-treated groups [F_(4,35)_ = 16.071, P < 0.05, Fig. 1E]. Moreover, in stress condition, the telmisartan produced significantly different results than that of the losartan, a selective AT receptor antagonist (p < 0.05). Overall, telmisartan showed a slightly smaller anti-depressant ability than fluoxetine, which is a selective serotonin reuptake inhibitor (SSRI) used to treat depression in clinics.

Furthermore, the PPARδ and 5-HTT expression levels in the hippocampus were significantly reduced in mice receiving UCMS. Chronic administration of telmisartan reversed the UCMS-induced changes in hippocampal PPARδ [F_(4,25)_ = 8.180, P < 0.05, Fig. 1E] and 5-HTT expressions [F_(4,25)_ = 6.662, P < 0.05, Fig. 1F] seen with Western blots. Losartan treatment did not affected PPARδ and 5-HTT expressions in UCMS mice. These results indicated that telmisartan effectively attenuates the adverse effect of stress on depression-like behavior, and this action seems different with other AT1 blockade, associated with increased PPARδ activation in the hippocampus.

### PPARδ antagonist inhibited the anti-depression-like effect of telmisartan in UCMS mice

**Figure 2:**
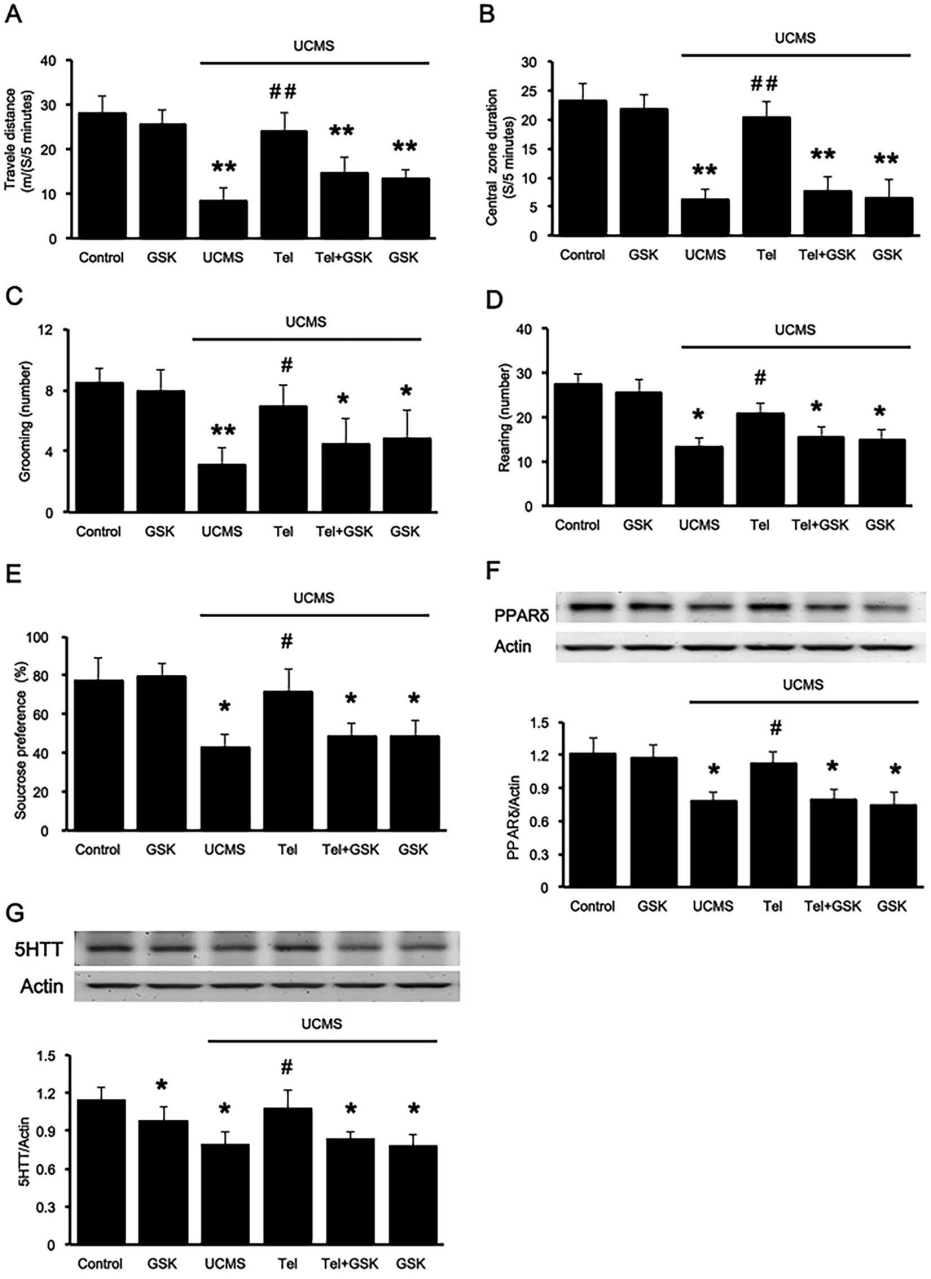
The PPARδ antagonist inhibited the anti-depression-like action of telmisartan in mice receiving UCMS. The PPARδ antagonist, GSK0660 (5 nmol.L^−1^, i.c.v), was administered 30 min before telmisartan treatment. In the open field test, the animals were allowed to explore the open field arena for 5 min. (**A**) the total distance travelled; (**B**) the time spent in the central; (**C**) frequency of grooming; (**D**) frequency of rearing; (**E**) the percentage of sucrose solution consumed (%) in the sucrose preference test (SPT); (**F**) PPARδ expression levels in the hippocampus of the various treatment groups; (**G**) 5-HTT expression levels in the hippocampus of the various treatment groups. Data are presented as the mean ± SEM (n = 8 per group). *p < 0.05 and **p < 0.01 compared to the corresponding control; ^#^p < 0.05 and ^##^p < 0.01 compared to the UCMS group. Full-length blots are presented in Supplementary Fig. S2.

For further investigation of the interaction between telmisartan and PPARδ in depression, PPARδ antagonist GSK0660 was administered into UCMS mice. Compared to the improvement in behavior observed in the telmisartan group, the total distance travelled [F_(5,42)_ = 49.285, P < 0.05], the time spent in the central [F_(5,42)_ = 46.881, P < 0.05], novelty-seeking behavior including rearings [F_(5,42)_ = 18.898, P < 0.05] and grooming [F_(4,35)_ = 26.573, P < 0.05] and percentile of sucrose preference [F_(5,42)_ = 54.628, P < 0.05] in the UCMS group co-treated with telmisartan and GSK0660 was markedly decreased. However, the results also showed that the administration of GSK0660 only in the UCMS groups did not further deteriorate the depression-like behavior (Fig. 2A,B,C,D,E).

Compared to the control group, the expression of hippocampal PPARδ was significantly reduced in mice receiving UCMS, and this was reversed by telmisartan. Once PPARδ was blocked by GSK0660, the effect was extinguished [F_(5,42)_ = 6.349, P < 0.05]. Likewise, a similar change in hippocampal 5-HTT expression [F_(5,42)_ = 5.864, P < 0.05] was observed [Fig. 2F,G]. Aditionally, the treatment of GSK0660 decreased 5-HTT expression but did not affect behavior performances in control group. Taken together, the anti-depressive effect of telmisartan is likely to be PPARδ-dependent. Additionally, administration of GSK0660 alone in mice receiving UCMS did not cause any changes in behavioral performance or expression levels compared to that observed in the vehicle-treated group.

### The anti-depressive effect of telmisartan disappeared in mice with hippocampal PPARδ knockdown

**Figure 3:**
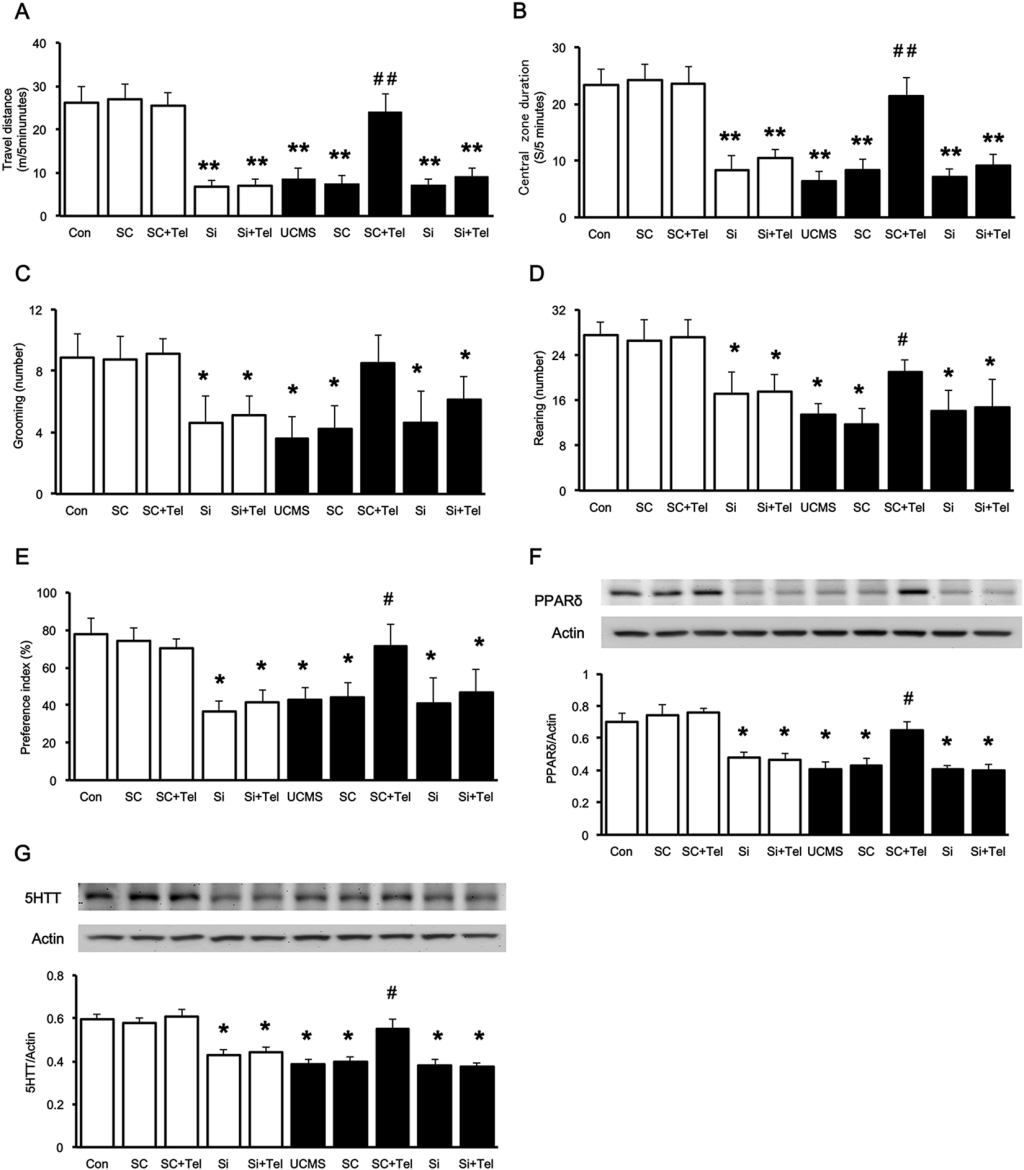
Changes of telmisartan-induced effects in the mice received hippocampal PPARδ knockdown. Normal mice and UCMS mice were transfected with scrambled shRNA or PPARδ shRNA, respectively. In the open field test, the animals were allowed to explore the open field arena for 5 min. (**A**) The total distance travelled; (**B**) the time spent in the central; (**C**) frequency of grooming; (**D**) frequency of rearing; (**E**) the percentage of sucrose solution consumed (%); (**F**) PPARδ expression levels in the hippocampus; (**G**) 5-HTT expression levels in the hippocampus. White columns indicated normal control goups, and black columns indecated UCMS goups. Data are presented as the mean ± SEM (n = 8 per group). *p < 0.05 and **p < 0.01 compared to the corresponding control; ^#^p < 0.05 and ^##^p < 0.01 compared to the UCMS group. Full-length blots are presented in Supplementary Fig. S3.

Then, we knocked down the expression of PPARδ using shRNA constructs in control and UCMS groups respectively. On the 7th day after the transfection of shRNA into the brain, decreased PPARδ expression in the hippocampus was identified. One week later, mice were treated following the procedure noted above. Changes in behavioral performance were then compared with that in animals injected with a scramble control. Mice with PPARδ knockout in hippocampus displayed a significantly decreased the total didtance of travelled and the time spent in the central. When PPARδ is silenced in control mice, telmisartan could not reverse the inactivated behavior (Fig. 3A,B,C,D,E]. Two-way ANOVA indicated telmisartan effects were dependent of the stress condition [effect of group, F_(4,70)_ = 81.419, P < 0.05; effect of stress, F_(1,70)_ = 338.827, P < 0.05; effect of group-by-stress interaction, F_(4,70)_ = 53.853, P < 0.05]. Moreover, telmisartan increased the grooming and rearing in the OFT and the sucrose consumption in the SPT in the scramble group but not in that of the PPARδ knockdown animals only in UCMS mice [grooming: effect of group, F_(4,70)_ = 15.791, P < 0.05; effect of stress, F_(1,70)_ = 23.421, P < 0.05; effect of group-by-stress interaction, F_(4,70)_ = 10.695, P < 0.05; rearing: effect of group, F_(4,70)_ = 22.557, P < 0.05; effect of stress, F_(1,70)_ = 65.24, P < 0.05; effect of group-by-stress interaction, F_(4,70)_ = 10.155, P < 0.05; and glucose consumption: effect of group, F_(4,70)_ = 15.345, P < 0.05; effect of stress, F_(1,70)_ = 60.92, P < 0.05; effect of group-by-stress interaction, F_(4,70)_ = 8.528, P < 0.05].

**Figure 4:**
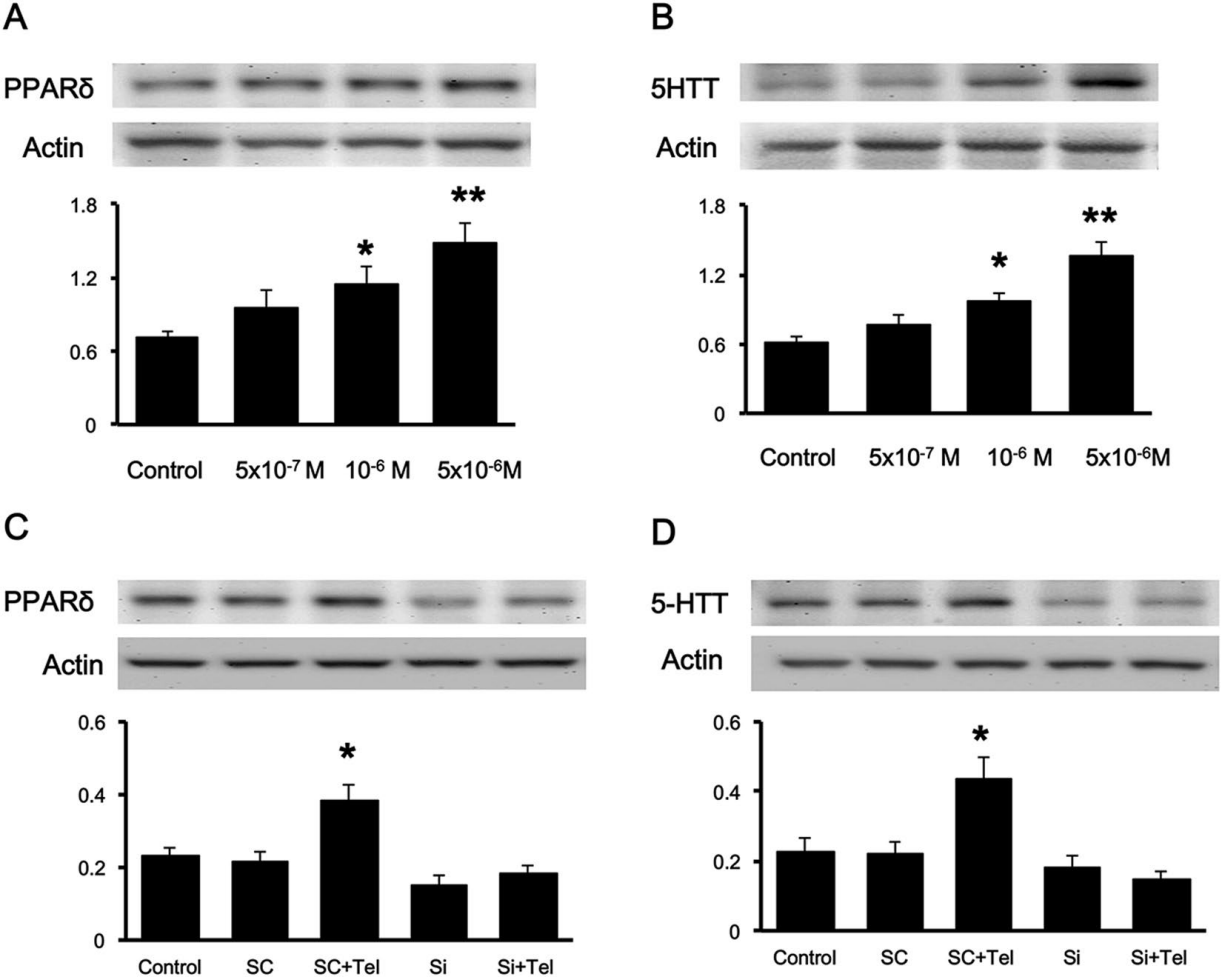
Effect of telmisartan on PPARδ and 5-HTT expression levels in cultured H19-7 cells. Cultured hippocampal H19-7 cells were incubated with different doses of telmisartan at 5 × 10^−7^ M, 10^−6^ M, and 5 × 10^−6^ M for 24 h. (**A**) PPARδ expression level and (**B**) 5-HTT expression level. Cells were transfected with scramble shRNA (Sc) or PPARδ shRNA (Si) for 24 h and then treated with 5 × 10^−6^ M telmisartan for 24 h. (**C**) PPARδ expression level and (**D**) 5-HTT-expression level. Each column represents the mean ± SEM (n = 6). *p < 0.05 and **p < 0.01 compared to the corresponding control. Full-length blots are presented in Supplementary Fig. S4.

Furthermore, the reduction in the PPARδ levels in the hippocampus of scramble mice was reversed by telmisartan only in UCMS group [effect of group, F_(4,50)_ = 13.325, P < 0.05; effect of stress, F_(1,50)_ = 79.649, P < 0.05; effect of group-by-stress interaction, F_(4,50)_ = 3.232, P < 0.05; Figs 3E, 4E]. Similar to the changes in PPARδ, the lower 5-HTT levels in the scramble group were also reversed after telmisartan treatment [effect of group, F_(4,50)_ = 15.277, P < 0.05; effect of stress, F_(1,50)_ = 35.710, P < 0.05; effect of group-by-stress interaction, F_(4,50)_ = 3.589, P < 0.05; Figs 3F, 4G]. However, telmisartan failed to restore the PPARδ and 5-HTT expression levels in the PPARδ knockdown mice with or without UCMS. It indicated that PPARδ deficiency correlated with the development of depression. The effects of telmisartan in the improvement of depression induced by UCMS seem to be associated with the selective enhancement of PPARδ and 5-HTT expression levels.

### Effect of telmisartan on blood pressure in UCMS mice

The mean systolic blood pressures in the vehicle-treated normal control group, telmisartan-treated normal group, UCMS + vehicle control group, and UCMS + telmisartan group were calculated at before, and the end of telmisartan treatment. As shown in Table 1, no statistical difference (P > 0.05) can be obtained in telmisartan-treated group at before and after stage, as that in vehicle-treated control (Table 1). Therefore, the behavioral effect of telmisartan seems to not be related to its antihypertensive action.

**Table 1:** Changes in systolic blood pressure of mice receiving telmisartan treatment.

Groups	Stage for Treatment	
	Before	After
Control	84.92 ± 2.94	84.04 ± 2.32
UCMS + Vehicle	82.54 ± 2.03	85.88 ± 2.95
UCMS + Telmisartan	81.42 ± 1.67	81.67 ± 2.88

Data (mean ± SEM, *n* = 8) show the value of systolic blood pressure (mmHg). Telmisartan was treated at 1 mg.kg^−1^ and the solvent used to disslove telmisartan was treated at same volume in vehicle-treated group.

### Telmisartan increased PPARδ and 5-HTT expressions in the H19-7 cell line

To understand the direct effect of telmisartan on PPARδ and 5-HTT expression levels, H19-7 cells were treated with telmisartan (3 × 10^−7^ mol.L^−1^, 10^−6^ mol.L^−1^ and 3 × 10^−6^ mol.L^−1^) or vehicle (control) for 24 h. The results showed that telmisartan increased PPARδ expression in a concentration-dependent fashion (Fig. 4A). Increased expression of 5-HTT was also observed in cells treated with telmisartan in the same manner (Fig. 4B). However, 5-HTT levels were not increased in PPARδ-silenced cells, even when they were treated with telmisartan (Fig. 4C,D). These results show that PPARδ is able to regulate 5-HTT expressions. Therefore, telmisartan failed to affect 5-HTT expressions when PPARδ was absent in hippocampal cells.

## Discussion

In the present study, we found that telmisartan is useful for alleviating the symptoms of depression. BALB/c mice are known to exhibit depressive-related behaviors when subjected to selected stress paradigms, offer much promise for the study of the stress response, and are good models for depression and the antidepressant treatment response in humans^22^. UCMS has been widely used in mice to mimic a depression-like disorder and is recognized as a reliable model of depression in humans^23^. Mice were exposed to UCMS and exhibited significant depressive behaviors, as shown by the decreased locomotor activity and suppressed grooming and rearing behavior in the OFT and reduced sucrose intake in the SPT. Additionally, food and water deprivation were applied in SPT, as described previously^24,25^. Chronic treatment with telmisartan significantly ameliorated the depressive-like behaviors in the chronic stress mice. Telmisartan effectively reversed the changes in the locomotor activity and frequency of grooming and rearing in the OFT and increased sucrose intake in the SPT. We have previously conducted some preliminary experiments and found no effect of telmisartan itself in normal mice. However, the anti-depressive action of telmisartan was still less than that of fluoxetine. In addition, telmisartan at the treated dosage did not affect blood pressure in mice that received UCMS. This is consistent with a previous report that reported that telmisartan at a non-hypotensive dose had beneficial effects on cognitive impairment^26^. A non-hypotensive dose of telmisartan preferentially promoted the expression of 5-HTT by activating PPARδ. It possibly because the wide distribution of PPARδ receptors in the brain, particularly the hippocampus.

Moreover, inhibition of brain AT1 receptor activity was approved to reduce stress responses and anxiety^27^. ARBs might have neuroprotective effect in addition to slow the progression of Alzheimer’s disease^28^. The antidepressant-like effect of ARBs has also been previously reported in depressed patients^29^. Additionally, our data showed that telmisartan was more effective on ameliorating depression-like behaviors in UCMS mice as compared with losartan. It means that telmisartan might act different with the other ARBs in depression treatment. Telmisartan can block cerebral AT1 receptors, which have a higher expression in the brain^30^. In contrast to classical ARBs, telmisartan is able to cross BBB^31^. Peripheral administration of losartan was less effective on cognitive function compared with telmisartan in diabetic mice^32^. However, directly injection of losartan into the amygdala showed anxiolytic-like effect in acute stressed rats^33^. Therefore, our results indicated that the absence of efficacy of losartan to counteract the UCMS effect is also probably due to the lack of BBB permeability.

Furthermore, ligands of PPARδ are known to interfere with 5-HTT signaling^34^. After a UCMS challenge, PPARδ and 5-HTT expression levels were both markedly reduced in the hippocampus as shown in the present study, which is in agreement with previous reports^34,35^. The levels of PPARδ and 5-HTT were effectively up regulated by telmisartan in the model group, indicating that the antidepressant-like action of telmisartan seems to be related to increased expression of PPARδ or 5-HTT. Notably, the amelioration of the behavioral performance in the OFT and SPT, as well as hippocampal PPARδ and 5-HTT expression levels, induced by telmisartan was inhibited by a PPARδ antagonist, GSK0660. Furthermore, down regulation of PPARδ alone reproduced the phenotypes of the UCMS as indicated in our previous study^9^. In PPARδ knockdown mice with UCMS, the depression-like behavior was significantly higher than those mice infected with the scrambled shRNA. The anti-depressive effect of telmisartan also disappeared once hippocampal PPARδs were silenced. Therefore, PPARδ is involved in the behavioral performance of mice, and telmisartan can promote PPARδ expression to improve depression-like behaviors.

PPARδ shows a relatively high neuronal expression compared with that of the other PPAR subtypes, both PPARα and PPARγ^36^. It has been mentioned that activation of PPARδ induces oligodendrocyte differentiation and enhances neuronal differentiation in the peripheral nervous system^37,38^. Additionally, a decrease in 5-HTT activity in the presynaptic membrane has been identified in depression patients^38^. Furthermore, depressive behaviors have also been observed in 5-HTT-knockout mice^39^. 5-HTT is one of the major modulators of 5-HT neurotransmission as it determines the magnitude and duration of 5-HT signaling. In the present study, changes in 5-HTT expressions were associated with PPARδ expression in both stress-induced depression mice and PPARδ-silenced mice. Therefore, PPARδ seems to interact with 5-HTT in the hippocampus. In cultured H19-7 hippocampus cells, we also demonstrated that PPARδ activation by telmisartan could increase 5-HTT expressions.

Chronic stress is associated with oxidative stress and promotes the production of reactive oxygen species, resulting in impaired function in the central nervous system^40^. Telmisartan as a unique ARB with a partial PPARδ agonistic property; has been shown to be neuroprotective and improve cognitive decline by reducing the levels of interleukin and TNF-α^41–43^. A recent study indicated that treatment with telmisartan reduced the levels of proinflammatory mediators and ameliorated the depression-like behaviors in diabetes-induced depression rats^44^. In addition, PPARδ activation in hypertensive rats is considered to contribute to the protection against cognitive decline, although it also up-regulates the expression of brain-derived neurotrophic factor in the hippocampus^45^. These findings probably explain why oral administration of the PPARδ antagonist GSK0660 or administration of PPARδ shRNA into the brain completely blocked the actions of telmisartan both *in vivo* and *in vitro*.

## Conclusions

Overall, we provided promising and novel evidence that up-regulating hippocampal PPARδ by telmisartan results in an anti-depressive effect through the elevation of 5-HTT expressions. Our study suggests that hippocampal PPARδ is an important therapeutic target for depression. Telmisartan could be used for the development of treatments for depressive disorders in clinics.

## Materials and Methods

### Animals

A total of 142 male BALB/c mice weighing 22–30 g were obtained from the National Animal Center (Taipei, Taiwan) and maintained in the animal center of Chi Mei Medical Center (Tainan, Taiwan). The animals were housed 3–4 mice per cage on a 12/12-hr light/dark cycle with ad libitum access to food and water except during behavioral tests. Mice were introduced to the experiment room at least 1 h before the behavioral tests. This project was approved by the Institutional Animal Care and Use Committee of Chi Mei Medical Center (No. 105111531). All of the animal procedures were performed according to the Guide for the Care and Use of Laboratory Animals published by the US National Institutes of Health (NIH Publication No. 85-23, revised 1996).

### Treatment schedule

**Figure 5:**
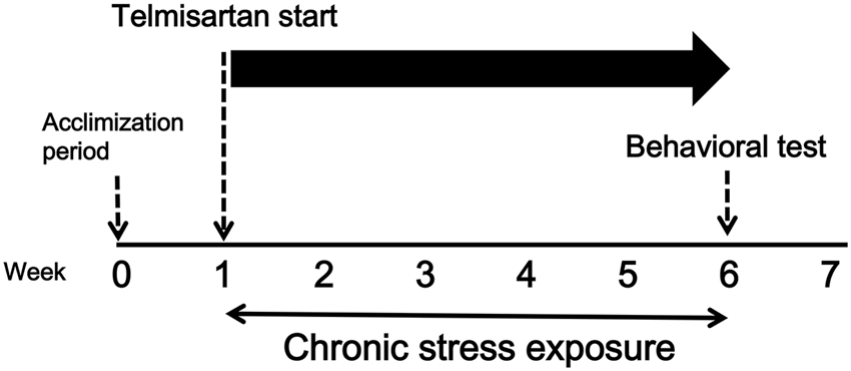
Timeline of experimental procedures.

This study was conducted according to the experimental protocols described in Fig. 5.

To investigate the effect of telmisartan on depression-like behavior in UCMS mice, mice were randomly divided into five groups (n = 8): control group, UCMS model group, UCMS + telmisartan (1 mg.kg^−1^) group^26^, UCMS + losartan (1 mg.kg^−1^) group, UCMS + fluoxetine (20 mg.kg^−1^) group^46^.

To investigate the role of telmisartan in PPARδ antagonist treated UCMS mice, mice were randomly divided into six groups (n = 8): control group, control group + GSK0660 (10 mg.kg^−1^), UCMS model group, UCMS + telmisartan (1 mg.kg^−1^) group, UCMS + GSK0660 (10 mg.kg^−1^) + telmisartan (1 mg.kg^−1^) group, and UCMS + GSK0660 (10 mg.kg^−1^).

To investigate the role of the role of telmisartan in PPARδ konckdown mice mice were randomly divided into 10 groups (n = 8): control, control + scramble, control + scramble + telmisartan, control + *PPARδ* shRNA, control + *PPARδ* shRNA + telmisartan, UCMS, UCMS + scramble, UCMS + scramble + telmisartan, UCMS + *PPARδ* shRNA, UCMS + *PPARδ* shRNA + telmisartan.

After an adaptation period (week 0 to week 1), mice were exposed to UCMS for 5 weeks (week 1 to week 6). Telmisartan, losartan and fluoxetine was administered 30 min by intragastric gavage before behavioral test and/or chronically 30 min before UCMS procedure to stressed as well as to unstressed control mice; while GSK0660 was administered by intraperitoneal injection, 30 min before drug treatment. All the animals were treated with respective drugs from week 1 to week 6. Behavioral testing was done in independent groups of mice in the week 7; all mice were subjected to one test daily, always in the same sequence. Blood pressure was measured every week during the experiment. Finally, all mice were sacrificed by cervical dislocation, and each hippocampus was removed, immediately frozen in liquid nitrogen, and kept at −80 °C for protein assays.

### The establishment of the depression-like mouse model

The UCMS model was used to explore depressive-like behaviors in mice as described previously^47,48^. Experimental mice (n = 8 per group) were exposed to unpredictable mild stressors randomly every day in one week. The stressors applied included the following: water deprivation (24 h), food deprivation (24 h), reversed light/dark cycle (24 h), overnight illumination (12 h), soiled cage (12 h), and cage tilt (18 h, 45°). Each stressor was randomly assigned two or three times over a 5-week period. Stressors continued to be applied during the testing phase, except on testing days to avoid effects of acute stress. The non-stressed control mice were housed in groups (3–4 per cage), and the stressed mice were singly housed^49^. At least 12 h of rest was provided between a stressor and a test^50^. All of the procedures were organized in a random in order to ensure the unpredictable characteristic of the experiment.

### Behavioral testing

#### OPT

Mice were placed in an open field area made of a 70 × 70 × 40 cm wooden box and equipped with an infrared floor to measure locomotor activity. The arena was subdivided into a central and a peripheral zone. Mice were placed in the open field boxes for 5 min under normal light conditions, and the locomotor activity of mice was automatically scored with a camera connected to a computerized system (Viewpoint, Lyon, France). Individual animals were gently placed in the same corner of the apparatus in all trials. Time stay in central, rearing (number of times the mice stood on their hind legs), grooming (total seconds of the mice spent licking or scratching itself) and excretion were observed^51^.

#### SPT

SPT is widely used to measure the anhedonic response, which was defined as a reduction in sucrose preference relative to baseline levels^52,53^. The mice were exposed to bottles, the one containing 1% sucrose and the other containing tap water for 24 h. After the deprivation of food and water overnight^24^, mice were used to receive the bottle of 1% (w/v) sucrose or the bottle of tap water for 1 hour. Then, the sucrose preference was evaluated according to the formula: sucrose preference = [sucrose intake/(sucrose intake + water intake)] × 100, as described previously^53^.

### Blood pressure measurement

To investigate the possible effect of telmisartan on blood pressure in depression-like mice, the systolic blood pressures were measured in mice received telmisartan treatment and others using the tail-cuff method by a sphygmomanometer without animal heating (Muromachi Kikai Co., Ltd., Tokyo, Japan). The blood pressure of mice under anesthesia was measured at 15-min intervals. Each value was calculated as the average of 3 measurements.

### Intracerebroventricular (ICV) injection

Mice were held in a towel with the dummy cannula to inject the testing agent as described previously^54^. Mice were anesthetized with a mixture of isoflurane in oxygen (2%) and placed in a Kopf stereotaxic instrument equipped with blunt ear bars. A dummy cannula was placed into the guide^55^. Mice were allowed to recover for 7 days.

The infusion cannula (22 gauge), attached to PE-10 tubing, was inserted into the guide cannula and extended 0.5 mm beyond the guide. A 10.0-μl Hamilton syringe was used to manually deliver saline or drugs over a two-minute period^56^. The infusion cannula was kept in place for an additional 1 min following infusion.

Moreover, the solution containing shRNA specific to PPARδ (Gene ID 25682) with an expression vector (pCMV6-Entry) was administered via ICV injection into mice using 25 μl of the prepared solution (0.12 μg.μl^−1^), while mice receiving a similar injection of an empty vector at the same volume were used as a control.

### Cell Cultures

Rat-derived hippocampus H19-7 cell line cells (CRL-2526; American Type Culture Collection, Manassas, VA) were maintained at 37 °C and 5% CO_2_ in Dulbecco’s modified Eagle’s medium (DMEM; HyClone, South Logan, UT, USA) with 4 mM l-glutamine that was adjusted with sodium bicarbonate (1.5 g/L), glucose (4.5 g/L), G418 (200 μg/mL), and puromycin (1 μg/mL) and supplemented with 10% fetal bovine serum^57^. Cells (1 × 10^6^) were plated on 60-mm culture dishes, and at 80% confluence, they were differentiated by culturing for 6–7 days in DMEM containing 2% fetal bovine serum. Medium was changed every other day.

### Western Blotting Analysis

Western blotting analysis was performed as previous^58^. Total protein lysates from mouse hippocampus or cells were extracted in lysis buffer (1% Triton X-100, 150 mM NaCl, 10 mM Tris [pH 7.5] and 5 mM ethylenediaminetetraacetic acid), containing a protease and phosphatase inhibitor cocktail (Sigma-Aldrich, MO, USA). The protein concentration was determined with the BCA assay kit (Pierce Biotechnology, Rockford, IL, USA). The following primary antibodies were used at 4 °C overnight: anti-PPARδ (1:1000) (Abcam, Cambridge, UK); anti-5-HTT (1:1000) (Merck Millipore, Darmstadt, Germany); anti-β actin (1:5000) (Merck Millipore) was used as an internal control. The next day, the blots were incubated with a 1/5000 dilution of horseradish peroxidase-conjugated secondary antibodies at 25 °C for 1 h. Protein bands were visualized using the enhanced chemiluminescence kit (PerkinElmer, Boston, MA, USA). The optical densities of the bands were determined using software (Gel-Pro Analyzer version 4.0 software; Media Cybernetics Inc., Silver Spring, MD, USA).

### Statistical analysis

Data were expressed as the mean-standard error of the mean. Statistical analyses one-way ANOVA was used to investigate the differences between groups with pharmacological treatments. Among multiple groups were analyzed by two-way ANOVA with “stress” and “drugs” are the factors to evaluate data in the knockdown experiments. If an interaction and/or main effect were observed, pairwise comparisons following ANOVA were made using Bonferroni post-hoc test. Data sets of two sample groups were analyzed using independent Student’s t-tests. All analyses were carried out by SPSS, version 21. Statistical differences were accepted at p < 0.05.

## Electronic supplementary material

Supplementary information
