# Supplementary material for: Telmisartan Activates PPARδ to Improve Symptoms of Unpredictable Chronic Mild Stress-Induced Depression in Mice
Source: Sci Rep. 2017 Oct 25;7:14021. doi: 10.1038/s41598-017-14265-4 (PMC5656622; doi:10.1038/s41598-017-14265-4)

Supplementary Information

Telmisartan Activates PPARδ to Improve Symptoms of Unpredictable Chronic Mild Stress-Induced Depression in Mice

Yingxiao Li[a,b], Kai Chun Cheng[a], Keng-Fan Liu[c], Wen-Huang Peng[c], Juei-Tang Cheng[b,d*], Ho-Shan Niu[e*]

Supplementary Fig S1 The full-length blots used in Fig. 1F, Fig. 1G.

Fig. 1F PPARδ

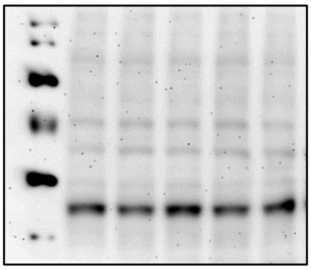

Fig. 1F Actin

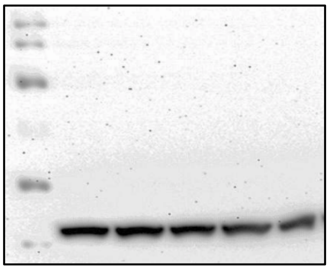

Fig. 1G 5-HTT

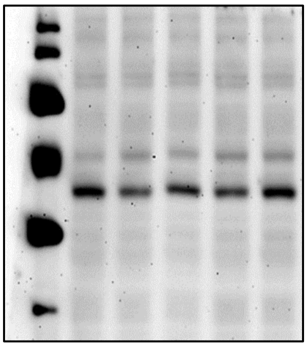

Fig. 1G Actin

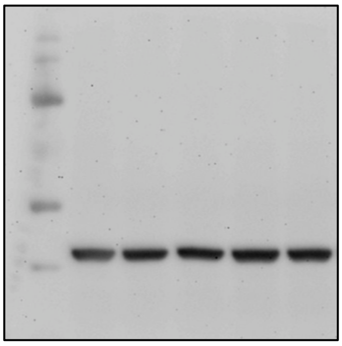

Supplementary Fig S2 The full-length blots used in Fig. 2F, Fig. 2G.

Fig. 2F PPARδ

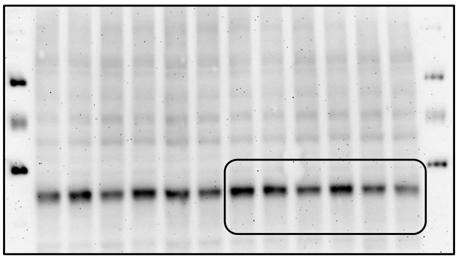

Fig. 2F Actin

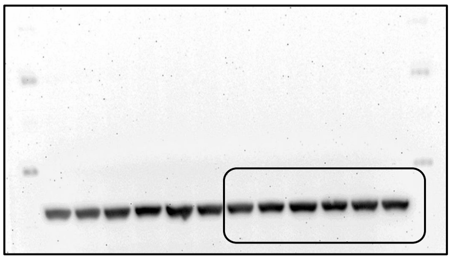

Fig. 2G 5-HTT

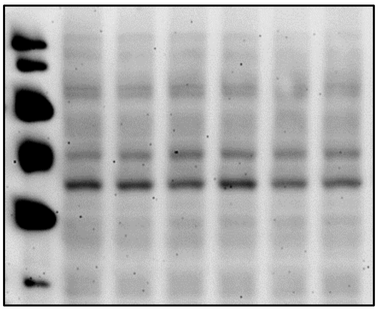

Fig. 2G Actin

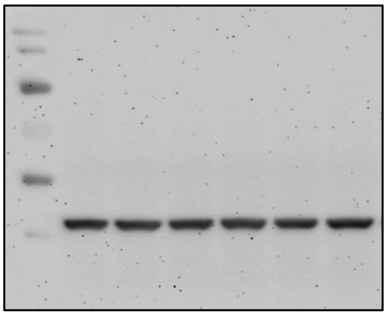

Supplementary Fig S3 The full-length blots used in Fig. 3F, Fig. 3G.

Fig. 3F PPARδ

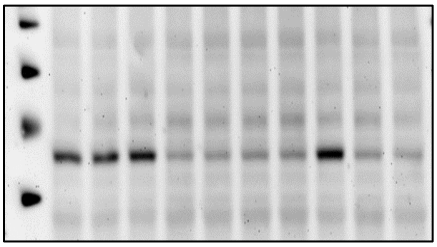

Fig. 3F Actin

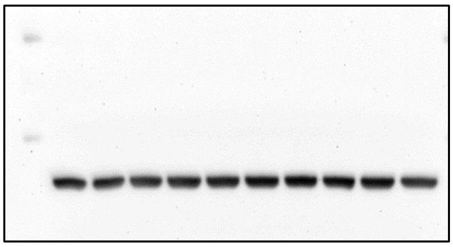

Fig. 3G 5-HTT

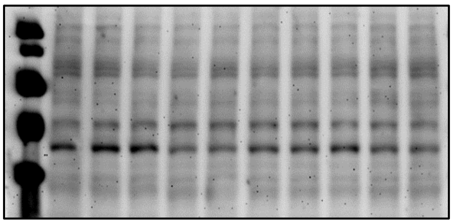

Fig. 3G Actin

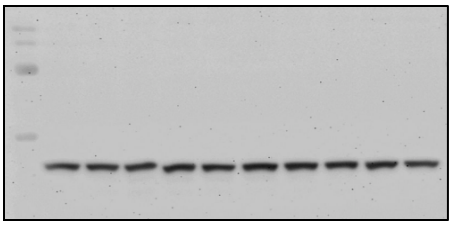

Supplementary Fig S4 The full-length blots used in Fig. 4A, Fig. 4B, Fig. 4C, Fig. 4D.

Fig. 4A PPARδ

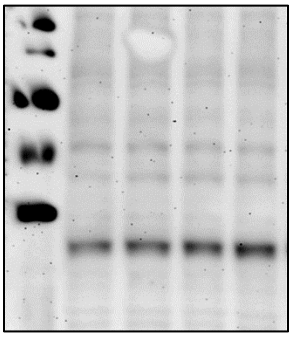

Fig. 4A Actin

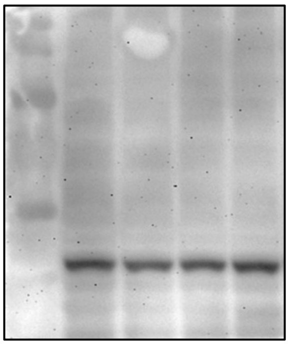

Fig. 4B 5-HTT

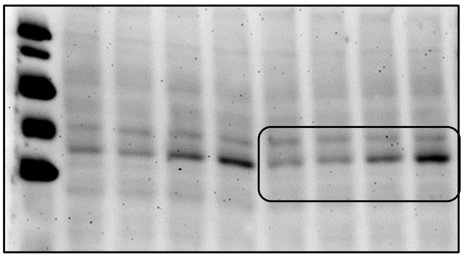

Fig. 4B Actin

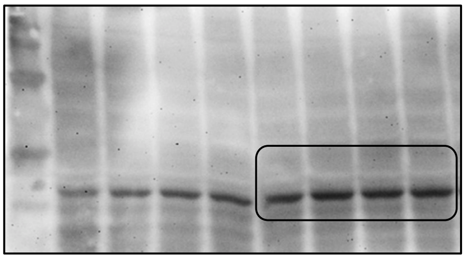

Fig. 4C PPARδ

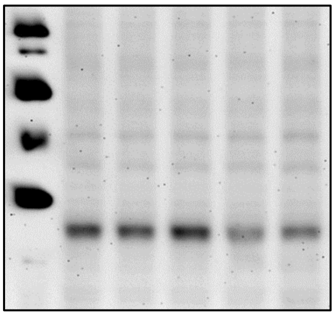

Fig. 4C Actin

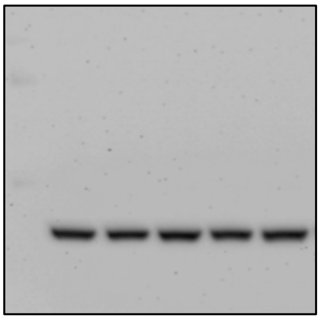

Fig. 4D 5-HTT

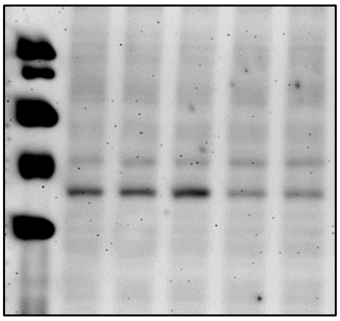

Fig. 4D Actin

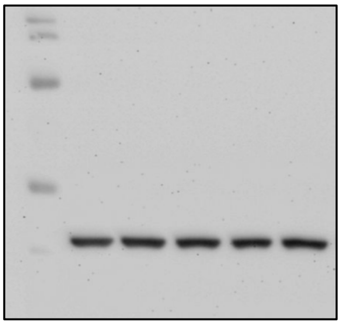

Supplement: Supplementary file 1 — Supplementary information [file 41598_2017_14265_MOESM1_ESM.pdf]
